# Asymmetric adult-onset asthma with periocular xanthogranuloma (AAPOX) associated with IgG4-related disease: a case report

**DOI:** 10.3389/fsurg.2025.1753451

**Published:** 2026-01-13

**Authors:** Qiaomei Huang, Zijian Chen

**Affiliations:** 1 Department of Ophthalmology, Meizhou People’s Hospital, Meizhou, Guangzhou, China; 2 The Affiliated Eye Hospital, Jiangxi Medical College, Nanchang University, Nanchang, Jiangxi, China

**Keywords:** AAPOX, asymmetric presentation, case report, corticosteroid therapy, IgG4-RD

## Abstract

**Background:**

Adult orbital xanthogranulomatous disease is a rare non-Langerhans cell histiocytosis, among which the adult-onset asthma with periocular xanthogranuloma (AAPOX) subtype is particularly uncommon. Recent studies have suggested an association between this condition and IgG4-related disease (IgG4-RD), possibly falling within its disease spectrum.

**Case presentation:**

A 60-year-old male presented with a one-year history of bilateral eyelid swelling accompanied by difficulty in opening the right eye. Examination revealed significant swelling and bulging of the right upper eyelid, with a well-defined, cord-like mass palpable on palpation. The left eyelid showed mild swelling without a detectable mass. The patient had a documented history of asthma, a positive bronchial provocation test, and significantly elevated serum IgE levels. Surgical intervention was performed on the right eye, followed by postoperative glucocorticoid therapy. Histopathological findings were consistent with xanthogranuloma, and IgG4 positivity suggested an association with IgG4-related disease (IgG4-RD). Serum IgG4 levels further supported this association. After treatment, the patient's serum IgG4 levels normalized, eyelid morphology improved significantly, and no progression was observed during a two-year follow-up period.

**Conclusion:**

This case further confirms the strong association between AAPOX and IgG4-RD, and represents the first report of asymmetric AAPOX. Surgical excision combined with systemic glucocorticoid therapy proved effective for this condition. Furthermore, the article explores the potential role of IgG4 produced during asthma desensitization therapy in the pathogenesis of IgG4-RD, suggesting that this potential risk should be considered during asthma treatment. This report provides valuable clinical experience for the diagnosis and treatment of such rare diseases.

## Introduction

Adult orbital xanthogranulomatous disease is a rare non-Langerhans cell histiocytosis, characterized by chronic inflammatory changes with histiocytic infiltration (1). It primarily affects the skin and subcutaneous tissues of the orbit and ocular adnexa, often presenting clinically as symmetric periorbital swelling (2). Its rarity results in limited understanding and a lack of well-defined treatment approaches (3). Adult orbital xanthogranuloma can be classified into four subtypes: adult-onset xanthogranuloma (AOX), necrobiotic xanthogranuloma (NBX), Erdheim-Chester disease (ECD), and adult-onset asthma with periocular xanthogranuloma (AAPOX) (1). Among these, the AAPOX subtype is particularly uncommon (4). AAPOX is typically characterized by yellow to orange cutaneous papules and nodules around the eyes, often accompanied by lymphadenopathy and adult-onset asthma (5).

In recent years, literature has suggested an association between adult orbital xanthogranuloma and IgG4-related disease (IgG4-RD), positing that it may fall within the spectrum of IgG4-RD (4, 6). IgG4-RD is a systemic immune-mediated condition of unknown etiology, featuring inflammation and tissue fibrosis. Its distinctive histopathological characteristics include lymphoplasmacytic infiltration rich in IgG4-positive plasma cells, sclerosing fibrosis, and obliterative phlebitis (7). Here, we report a rare case of asymmetric AAPOX associated with IgG4-RD and provide a detailed discussion of our therapeutic strategy, hoping to offer guidance for the future clinical management of AAPOX.

## Case presentation

**Figure 1:**
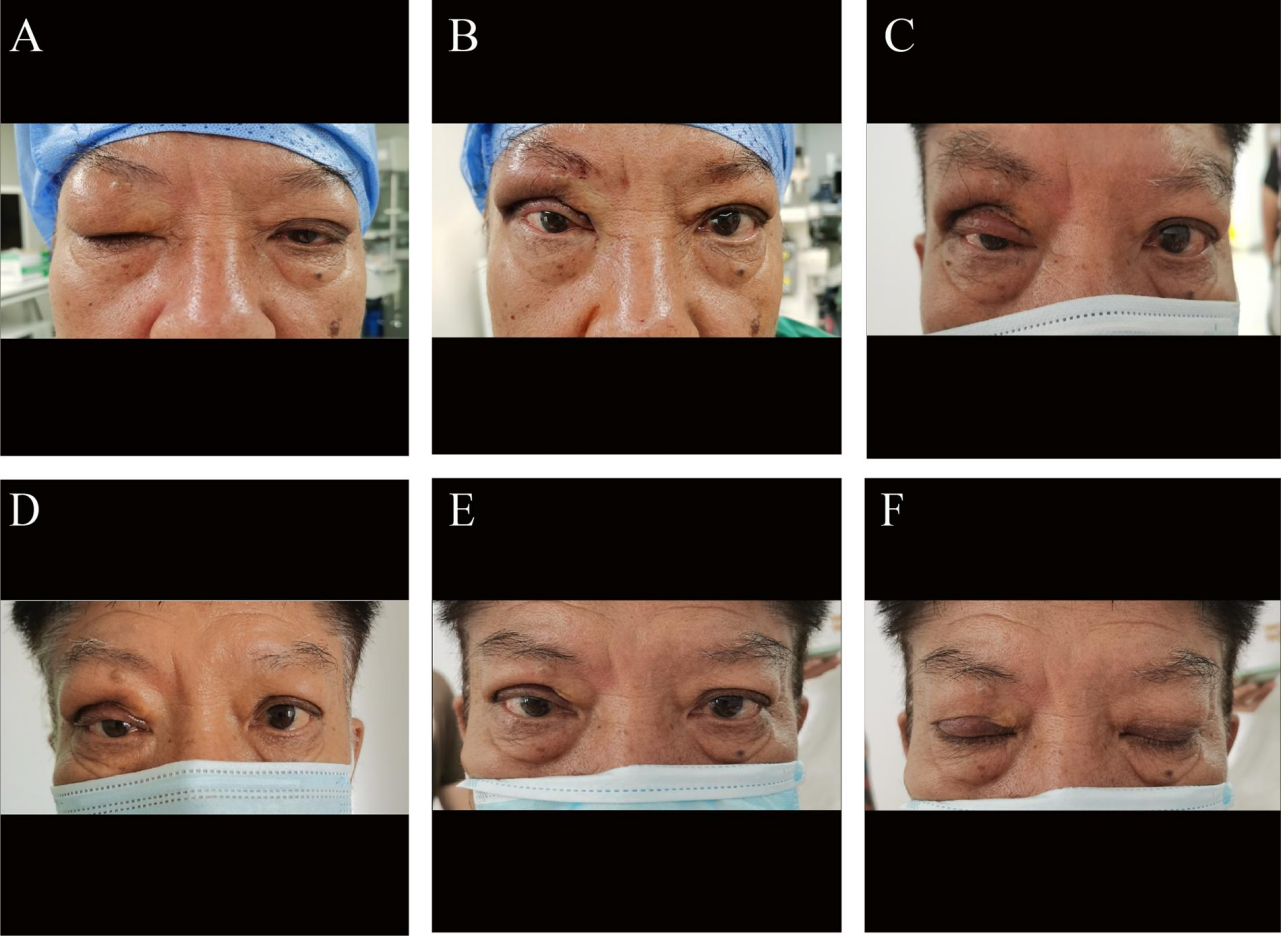
Clinical photographs of the eyelids before and after treatment. **(A)** Preoperative appearance showing significant swelling and mechanical ptosis of the right upper eyelid, with mild edema of the left upper eyelid. **(B)** Immediate postoperative appearance after right orbital mass excision and blepharoplasty. **(C,D)** Postoperative appearance at one week and one month, respectively, demonstrating significant improvement in right eyelid morphology and reduction of ptosis. **(E,F)** Postoperative appearance at 3 months, with eyes open **(E)** and closed **(F)**, showing stable and satisfactory outcome.

A 60-year-old male patient presented to our hospital with a one-year history of progressively worsening swelling of both upper eyelids, accompanied by difficulty in opening the right eye. Significant swelling and bulging of the right upper eyelid with mechanical ptosis were observed, while the left upper eyelid showed mild edema (Figure 1A). Examination revealed a visual acuity of 20/20 in both eyes, with intraocular pressure within normal ranges. A well-defined, cord-like mass measuring approximately 3 cm × 1 cm was palpated along the superior orbital rim of the right eye. The mass was firm, mobile, non-tender, and without surface ulceration or bleeding. No mass was detected in the left eye. Notably, further history revealed the patient had experienced an asthma attack one year prior, for which he underwent three months of desensitization therapy. He was not currently taking asthma medication regularly.

**Figure 2:**
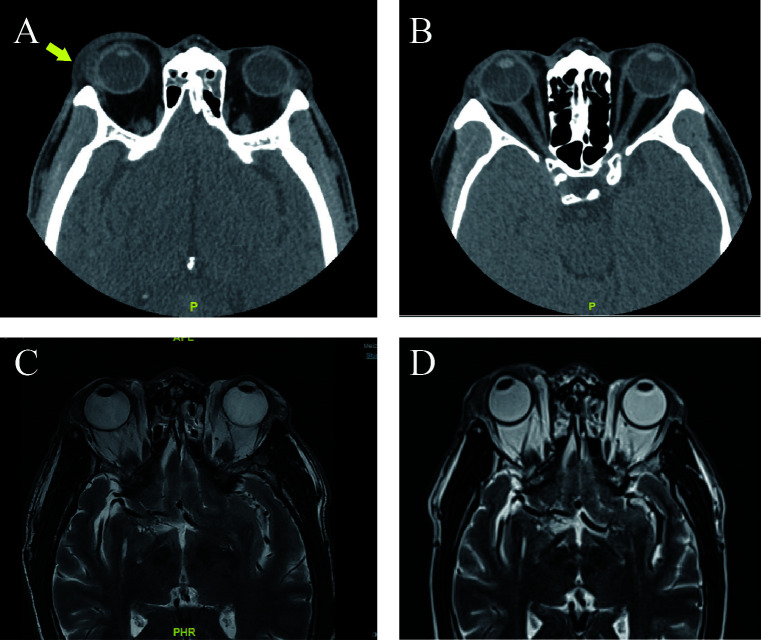
Radiological imaging of the orbits. **(A)** Preoperative axial computed tomography (CT) scan revealing soft tissue swelling and a small nodule (arrow) in the medial aspect of the right upper eyelid. **(B)** Postoperative axial CT scan at 3 days confirming successful removal of the right orbital mass. **(C)** One-month postoperative coronal T2-weighted magnetic resonance imaging (MRI) showing bilateral lacrimal gland and surrounding soft tissue swelling. **(D)** Postoperative coronal T2-weighted MRI at 2 years showing no evidence of disease progression.

**Figure 3:**
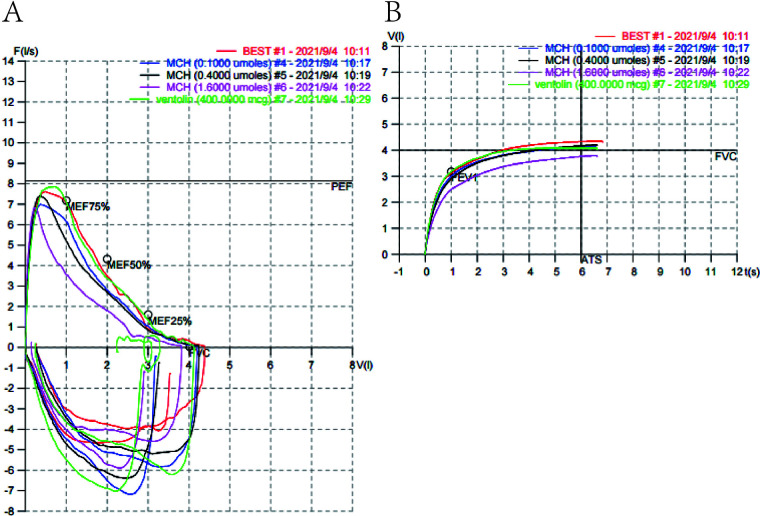
Pulmonary function test. **(A)** Flow-volume curve from the bronchial provocation test. **(B)** Time-volume curve from the bronchial provocation test. Both curves are consistent with a positive test, indicating airway hyperresponsiveness.

Subsequent orbital CT revealed swelling of the right upper eyelid with a small nodule visible medially; both globes appeared normal (Figure 2A). Given the patient's history of asthma, additional tests, including a bronchial provocation test (Figure 3) and serum IgE level, were performed, both of which yielded positive results (IgE: 1,520.00 IU/mL). Other laboratory findings, including complete blood count, liver and kidney function, and coagulation profile, were within normal limits.

**Figure 4:**
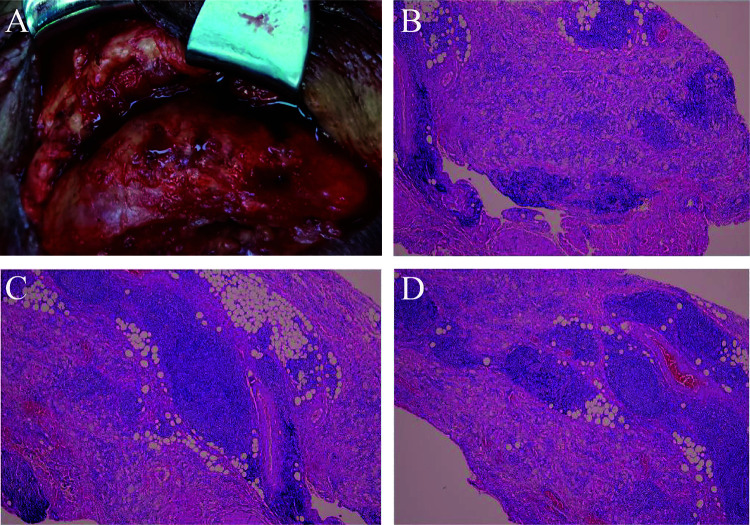
Intraoperative findings and pathological results. **(A)** Intraoperative view showing diffuse yellowish infiltration of the subcutaneous tissue and the well-defined mass; **(B–D)** histopathological examination (Hematoxylin and Eosin stain). Histiocytic and lymphocytic infiltration visible within the reticular dermis.

To clarify the nature of the mass, following discussion between the ophthalmology and respiratory departments, the patient underwent partial excision of the right orbital mass combined with blepharoplasty under local anesthesia. Intraoperative findings revealed diffuse yellowish tumor growth in the subcutaneous tissue, with thickened skin exhibiting yellowish discoloration and diffuse yellowish infiltration of the subcutaneous tissue. The lesion adhered to surrounding structures, involving the lacrimal gland, orbital septum, orbital fat, and levator palpebrae superioris muscle, with fibrosis and firm consistency in the affected areas (Figure 4A). The affected tissues and mass were resected as completely as possible while preserving the levator muscle. Part of the mass was sent for pathological examination. Postoperative CT confirmed successful removal of the right orbital mass (Figure 2B).

Histopathological and immunohistochemical results showed proliferation of lymphocytes, plasma cells, and histiocytes within the tissue. Staining was positive for CD38, CD138, CD68 (histiocytes), and IgG. A small number of IgG4-positive cells were observed, with an IgG4/IgG ratio of less than 40%. The Ki-67 proliferation index was approximately 15%. These findings were consistent with a diagnosis of xanthogranuloma and highly suggestive of an association with IgG4-RD. This led us to consider a probable diagnosis of AAPOX (Figures 4B–D). Since AAPOX often presents with bilateral symmetric involvement, a postoperative orbital MRI was performed, which revealed abnormal changes in the bilateral lacrimal glands and surrounding upper eyelid-frontal soft tissues, more pronounced on the right side (Figure 2C). This confirmed that the left upper eyelid was also affected, albeit to a much lesser degree than the right.

To further clarify the association with IgG4-RD, the patient's serum IgG4 level was measured postoperatively and was found to be significantly elevated. Based on our clinical experience, oral glucocorticoid therapy (methylprednisolone tablets) was initiated post-surgery, starting at 40 mg per day, with a weekly taper of 4 mg. The treatment regimen was adjusted based on the patient's tolerance and side effects during follow-up. Over a 6-month follow-up period, the serum IgG4 level progressively decreased to within the normal range (Table 1). Postoperatively, the right eyelid morphology showed significant improvement (Figures 1B–D), and follow-up imaging studies showed no evidence of disease progression (Figures 2B–D).

**Table 1:** Postoperative glucocorticoid dosage and serum IgG4 levels.

Methylprednisolone daily dose (mg/day)	Duration (days)	Serum IgG4 level (g/L)	Reference range (g/L)	Examination time (postoperative)
40	7	11.80	0.03–2.01	One month
36	7			
28	7	2.43		Three months
20	7			
16	14	1.65		Six months
12	14			
4	14			

Two weeks after starting oral corticosteroids, the patient developed insomnia, hand twitching, and irritability. We therefore adjusted the tapering schedule and duration of treatment (total treatment duration: 70 days).

## Discussion and conclusions

Adult orbital xanthogranulomatous disease has a low incidence rate, and the AAPOX subtype presented in this case is particularly rare. Currently, no standardized diagnostic and therapeutic guidelines exist (3). In this case, we did not suspect AAPOX preoperatively and proceeded with diagnostic surgical intervention based on a preliminary diagnosis of an orbital mass. Consequently, the preoperative workup had certain limitations, such as the lack of an orbital MRI, serum IgG4 levels, and lymph node assessment. During the surgery, the intraoperative findings and subsequent postoperative pathological results raised a strong suspicion of AAPOX, prompting us to perform the relevant supplementary investigations. Furthermore, the patient had a history of asthma exacerbation within the past year, supported by a positive bronchial provocation test and significantly elevated serum IgE levels (1,520.00 IU/mL). These additional findings, combined with the patient's medical history, substantiated the diagnosis of AAPOX. This case underscores the importance for clinicians to proactively inquire about systemic history, particularly regarding asthma, in patients presenting with bilateral orbital tumors.The patient presented with significant swelling and elevation of the right upper eyelid accompanied by mechanical ptosis, complaining of an inability to open the right eye. To determine the nature of the mass and simultaneously address the ptosis, we performed a partial excision of the right orbital mass combined with blepharoplasty. Postoperatively, mild residual ptosis was observed in the right eye. This was primarily attributed to involvement of the levator palpebrae superioris muscle by the lesion, a conclusion supported by the intraoperative findings and the improvement in ptosis following glucocorticoid therapy.

The currently accepted diagnostic criteria for IgG4-RD are: (i) clinical examination showing characteristic diffuse/localized enlargement or masses in one or multiple organs; (ii) elevated serum IgG4 levels (≥1.35 g/L); (iii) histopathology revealing dense lymphoplasmacytic infiltration, fibrosis, and IgG4-positive plasma cell infiltration [IgG4-positive plasma cells/IgG-positive plasma cells >40%, and IgG4-positive plasma cells >10 per high-power field (HPF), or >50/HPF in the lacrimal gland], while excluding other autoimmune diseases and malignancies. A definitive diagnosis requires (i) + (ii) + (iii); a probable diagnosis requires (i) + (iii) or (i) + (ii) (7). Although the pathological findings in this case indicated hyperplasia of lymphocytes, plasma cells, and histiocytes, the IgG4/IgG ratio was <40%, which does not support criterion (iii). Therefore, this case meets criteria (i) and (ii), constituting a probable diagnosis. This further supports that AAPOX is highly likely to be a manifestation within the spectrum of IgG4-RD. IgG4, a subtype of IgG, has immunomodulatory properties. In asthma desensitization therapy, an increase in IgG4 is a marker of treatment efficacy (8). This is because chronic allergen exposure induces immune tolerance, one key mechanism being the induction of high levels of IgG4. These IgG4 antibodies compete with pathogenic IgE for binding to the same allergens, thereby preventing IgE from activating mast cells and other immune cells and averting allergic symptom onset (8). However, when IgG4 is produced in large quantities and infiltrates specific tissues (e.g., pancreas, lacrimal glands), it can likely trigger IgG4-RD (9). This leads to the consideration that excessive allergen exposure during the asthma course might have contributed to the onset of orbital xanthogranuloma in this case. Therefore, during asthma treatment, close attention and monitoring are warranted to prevent potential risks, potentially avoiding excessive desensitization therapy. It is worth noting that the patient in this case had undergone three months of desensitization therapy. The core mechanism of desensitization therapy is to induce immune tolerance through chronic allergen exposure, thereby stimulating regulatory B cells to produce high levels of IgG4 antibodies. These IgG4 antibodies can compete with pathogenic IgE for binding to the same allergens, thereby inhibiting the activation of immune cells such as mast cells and achieving the purpose of treating asthma. However, in this case, this therapeutically induced IgG4 immune response, intended to be protective, may have become dysregulated. We speculate that in individuals with specific genetic backgrounds or an intrinsic Th2-high immune phenotype, the potent elevation of IgG4 triggered by long-term allergen exposure (such as from desensitization therapy) might instead precipitate a systemic immune disorder. The extensively produced IgG4 antibodies and their associated lymphoplasmacytic cells may abnormally infiltrate and accumulate in specific tissues (such as the periorbital area), thereby initiating the pathological process of IgG4-RD, manifesting as AAPOX. Therefore, during asthma treatment, close attention and monitoring of the treatment process are essential for prevention, and excessive desensitization therapy should be avoided as much as possible.

In previous studies, common histopathological findings in AAPOX include a layer of mononuclear foam-like histiocytes accompanied by lymphoplasmacytic infiltration and Touton giant cells. Immunohistochemical analysis typically shows strong expression of CD68, CD163, and Factor XIIIa in the foam-like histiocytes, while being negative for CD21, CD35, S100, and CD1a (10). However, AAPOX is exceedingly rare, with fewer than 50 cases reported in the literature to date (11). Coupled with subtle inter-individual variations, this significantly increases the diagnostic difficulty. For instance, in the present case, the patient presented with significant swelling of the right upper eyelid but only mild swelling of the left upper eyelid, which distinctly differs from the more commonly described bilateral symmetric presentation. Therefore, we opted for a diagnostic excision of the orbital mass while aiming to alleviate the patient's symptoms maximally. Regarding pharmacological treatment, we chose oral glucocorticoids (Methylprednisolone tablets), starting at 40 mg per day and tapering by 4 mg weekly. Notably, the patient developed insomnia, hand tremors, and irritability two weeks after initiating steroid therapy, leading us to appropriately adjust the tapering schedule and total treatment duration (total course: 70 days). The outcomes confirmed the effectiveness of our treatment regimen, with the patient's serum IgG4 levels normalizing within six months and no recurrence observed over two years.

Currently, various treatment modalities have been employed for adult orbital xanthogranuloma with differing outcomes, including surgery, local glucocorticoid injections, systemic glucocorticoids, chemotherapeutic agents, and/or radiotherapy (3). For AAPOX, our experience demonstrates that the combination of surgical intervention and systemic glucocorticoid therapy is highly effective. Previous studies have also confirmed that intralesional corticosteroids are an effective and safe treatment for adult xanthelasma of the eyelids (12). Furthermore, the patient's serum IgG4 levels gradually decreased to normal following glucocorticoid administration, and no progression of eyelid swelling was observed in either eye during the two-year follow-up. Therefore, we consider the use of glucocorticoids necessary for controlling the progression of AAPOX. Regarding long-term management, the occurrence of side effects in our patient underscores the importance of considering steroid-sparing agents for maintaining remission, especially in cases requiring prolonged therapy or with contraindications to corticosteroids. Agents such as methotrexate, mycophenolate mofetil, or rituximab have shown efficacy in managing IgG4-RD and other inflammatory orbital diseases (13). Future management of AAPOX could benefit from a treatment strategy similar to that of IgG4-RD, utilizing corticosteroids for initial induction and introducing steroid-sparing immunomodulators for maintenance.

Although this case report provides valuable insights into the diagnosis and treatment of asymmetric AAPOX, it has several limitations. First, we reported on only a single patient, and the generalizability of the conclusions requires validation through the accumulation of more cases. Second, due to the extreme rarity of AAPOX, we were unable to establish a definitive diagnosis preoperatively. This led to an insufficient preoperative workup, such as the absence of preoperative orbital MRI and serum IgG4 testing. These crucial ancillary investigations were only supplemented postoperatively after obtaining the pathological results, which highly suggested AAPOX. While this reflects the genuine diagnostic challenges encountered in clinical practice with rare diseases, a more comprehensive preoperative evaluation system might have provided earlier diagnostic clues. Furthermore, the patient's glucocorticoid treatment course and regimen were adjusted based on individual response. As mentioned, the patient developed glucocorticoid-related side effects, necessitating adjustments to the originally planned tapering schedule. Although such individualized adjustment is routine in clinical practice, it means this was not a standardized treatment protocol. Its long-term efficacy and optimal management strategy still need to be determined by larger-scale studies. Finally, a two-year follow-up period may still be insufficient for assessing the long-term risk of recurrence in this chronic disease. Patients will require further follow-up in the future. Despite these limitations, we believe this case offers valuable real-world experience and insights for clinicians in recognizing and managing this rare disorder.

## Data Availability

The original contributions presented in the study are included in the article/Supplementary Material, further inquiries can be directed to the corresponding author.
